# In Vitro Activity of Rezafungin Against Planktonic and Biofilm Forms of *Candida albicans* and *Nakaseomyces glabratus* Clinical Isolates from Vascular Infections in Poland: A Pilot Study

**DOI:** 10.3390/pharmaceutics18020213

**Published:** 2026-02-08

**Authors:** Iwona Skiba-Kurek, Magdalena Namysł, Katarzyna Kania, Joanna Czekajewska, Anna Sepioło, Tomasz Gosiewski, Aldona Olechowska-Jarząb

**Affiliations:** 1 Department of Microbiology, University Hospital, Jakubowskiego 2, 30-688 Kraków, Poland; iskiba@su.krakow.pl (I.S.-K.); asepiolo@su.krakow.pl (A.S.); 2 Department of Pharmaceutical Microbiology, Faculty of Pharmacy, Jagiellonian University Medical College, Medyczna 9, 30-688 Kraków, Poland; ka.kania@uj.edu.pl (K.K.); j.czekajewska@uj.edu.pl (J.C.); 3 Laboratory of Microbiology, The St. John Paul II Specialist Hospital, Prądnicka 80, 31-202 Kraków, Poland; 4 Department of Molecular Medical Microbiology, Chair of Microbiology, Faculty of Medicine, Jagiellonian University Medical College, Czysta 18, 31-121 Kraków, Poland; tomasz.gosiewski@uj.edu.pl

**Keywords:** rezafungin, yeast-like fungi, echinocandins, biofilm, invasive fungal infection, candidaemia, antifungal resistance

## Abstract

**Background/Objectives**: Certain yeast species are recognized as significant opportunistic pathogens, capable of causing severe systemic infections, particularly in immunocompromised individuals or those with disrupted physiological barriers. The rising incidence of invasive candidiasis associated with vascular infections poses a significant clinical challenge due to the high mortality rates and the limited efficacy of conventional antifungal therapies. The formation of resilient biofilms on vascular catheters by species such as *Candida albicans* and *Nakaseomyces glabratus* further complicates treatment, often leading to persistent fungemia and necessitating device removal. With the emergence of multidrug-resistant (MDR) strains, there is a critical need for new therapeutic agents like rezafungin—a novel, long-acting echinocandin with potential enhanced antibiofilm activity. **Methods**: This study tested susceptibility to antimycotics available in Poland (fluconazole, voriconazole, posaconazole, amphotericin B, anidulafungin, caspofungin, and micafungin) using the commercial Micronaut-AM test (Bruker, Bremen, Germany). Susceptibility to rezafungin (Angene Chemical, Great Britain) was determined using the microdilution method in RPMI medium, recommended by European Committee on Antimicrobial Susceptibility Testing (EUCAST), with amphotericin B as a control compound. We evaluated the biofilm-forming capacity and the in vitro activity of rezafungin against 42 clinical isolates of *Candida albicans* and *Nakaseomyces glabratus* recovered from positive blood cultures. **Results**: The obtained minimum inhibitory concentration (MIC) values suggest rezafungin activity against all the tested isolates, with different susceptibility to echinocandins and other antifungal drugs (azoles, amphotericin B) currently registered and used in Poland. The MIC readings for rezafungin were in the range of 0.008–0.5, with MIC_50_ = 0.016 and MIC_90_ = 0.25. The isolates were categorized as low, moderate, or strong biofilm producers according to established Stepanović criteria (cut-off values OD_630_ < 0.019, 0.19–0.38, >0.38, respectively). Furthermore, the higher minimum biofilm eradication concentrations (MBECs) compared to the minimum inhibitory concentrations (MICs) of planktonic cells confirm the reduced activity of rezafungin against biofilms. **Conclusions**: Critically, the high antibiofilm efficacy at clinically achievable concentrations suggests that rezafungin shows promise as a potential therapeutic option for catheter-related candidemia, though further clinical studies are needed. Furthermore, the high susceptibility of *N. glabratus* isolates—a species frequently associated with azole resistance—suggests rezafungin may be a valuable addition to the existing antifungal arsenal of multidrug-resistant (MDR) fungal infections in hospital settings. Future research should focus on in vivo models to confirm if these in vitro results translate into accelerated clearance of vascular biofilms.

## 1. Introduction

Invasive fungal infections (IFIs), particularly candidemia and invasive candidiasis, represent a major public health challenge characterized by high morbidity and mortality rates [1]. Each year, approximately 1.9 million patients develop acute IFIs, with global mortality ranging from 10% to 49% [2]. The clinical management of these infections is increasingly complicated by the emergence of drug-resistant species, notably *Nakaseomyces glabratus* (formerly *Candida glabrata*) and *Candidozyma auris* (formerly *Candida auris*) [3].

Echinocandins, such as caspofungin, micafungin, and anidulafungin, remain the cornerstone of therapy for IFIs in both neutropenic and non-neutropenic patients [4,5]. However, the rise of multidrug-resistant (MDR) strains and the inherent ability of *Candida* to form resilient biofilms necessitate the expansion of our therapeutic arsenal. Rezafungin, a novel echinocandin recently approved by the European Medicines Agency (EMA) and by the US Food and Drug Administration (FDA), addresses several limitations of earlier agents [6,7]. As a structural analogue of anidulafungin, rezafungin features a unique choline moiety that enhances chemical stability and eliminates reactive intermediates, thereby reducing toxicity [8].

Rezafungin is distinguished by superior pharmacokinetic and pharmacodynamic (PK/PD) properties, including an extended half-life of approximately 80 h [9]. This profile allows for once-weekly dosing, improving tissue penetration and safety compared to daily echinocandins [10]. Like its predecessors, rezafungin inhibits 1,3-β-D-glucan synthesis, leading to osmotic instability and cell death [11]. Its spectrum of activity includes azole-resistant *Aspergillus* and echinocandin-resistant *C. auris* [12,13]. Notably, rezafungin demonstrates potent activity against both adherent and mature *Candida* biofilms, suggesting efficacy in both prevention and treatment [14].

The persistence of invasive candidiasis is often driven by biofilm formation on medical devices, such as central venous catheters and prostheses [15]. Biofilm development follows a four-stage process: (1) early adherence (up to 11 h); (2) intermediate proliferation (12–30 h), including hyphal networking in *C. albicans*; (3) maturation (38–72 h) with extracellular matrix accumulation; and (4) cell dispersion. Crucially, *N. glabratus* lacks true hyphae, forming a more compact biofilm that may exhibit different susceptibility patterns to agents like rezafungin. Despite its registration in 2023, rezafungin is not yet widely used in Poland, highlighting a critical need for local susceptibility data.

Understanding the pathophysiology of vascular infections requires distinguishing the unique characteristics of fungal pathogens from those of bacteria. Unlike prokaryotic bacteria, such as *Staphylococcus epidermidis*, which we have previously characterized as a major cause of vascular-related biofilms in Poland [16], *Candida albicans* is a eukaryotic organism. This eukaryotic nature presents a significant therapeutic challenge, as many cellular targets are shared with the human host, potentially increasing the risk of treatment-related toxicity. A hallmark of *C. albicans* is its morphological plasticity, or dimorphism—the ability to switch between yeast and hyphal forms. While bacteria like *S. epidermidis* primarily rely on specialized adhesion molecules and the production of polysaccharide intercellular adhesin (PIA) to establish infections, the hyphal form of *C. albicans* actively penetrates vascular endothelial cells through both induced endocytosis and physical force. Furthermore, fungal biofilms are structurally more complex than the predominantly single-species bacterial biofilms. They consist of an organized, multilayered architecture of yeast, pseudohyphae, and hyphae encased in an extracellular matrix rich in β-1,3-glucans and mannans. This matrix acts as a physical and chemical shield that sequesters antifungal agents, making fungal vascular infections particularly recalcitrant to standard antimicrobial therapy compared to bacterial ones. The aims of this study were as follows: (1) to evaluate the in vitro activity of rezafungin against 42 clinical isolates of *C. albicans* and *N. glabratus* obtained from patients with invasive fungal infections (IFIs) at the University Hospital in Krakow; (2) to compare the antifungal potency of rezafungin with that of conventional echinocandins (anidulafungin, caspofungin, and micafungin); and (3) to determine the impact of rezafungin on both the formation and eradication of fungal biofilms.

## 2. Materials and Methods

### 2.1. Patients, Study Samples, and Strain Characteristics

This study was conducted on *C. albicans* (*n* = 27) and *N. glabratus* (*n* = 15) isolates collected from adult patients hospitalized at the University Hospital in Krakow, Poland, between XI 2023 and XI 2024. Clinical strains were isolated from bloodstream infections (venous, arterial, and catheter-drawn blood) from 42 patients hospitalized in various departments (Supplementary Materials, Table S1). Classic culture methods were used to detect the yeast presence in blood. The first stage of culture consisted in incubating the blood collected from each patient at 37 °C, using an automatic system for detecting microbiological infections (BacT/ALERT 3D 480, BacT/ALERT VIRTUO CLINIC, bioMérieux France, Marcy-l’Étoile, France). After the culture device indicated a positive culture, it was carried out on Sabouraud gentamicin chloramphenicol 2 agar (bioMérieux, Warsaw, Poland) at 35 °C and then isolated on Sabouraud dextrose agar (bioMérieux, Warsow, Poland) for further identification and drug susceptibility testing. The strain identification was performed using matrix-assisted laser desorption/ionization time-of-flight mass spectrometry (MALDI-TOF MS; Bruker Daltonics, Bremen, Germany) with the VITEK^®^ MS system (bioMérieux, Marcy-l’Étoile, France, software version V3.2). The strains were stored at minus 70 °C for further research.

International standard *Candida* species were used as quality control (QC) for the tested isolates.

The assay was also performed for reference strains recommended by the test manufacturer: *Candida parapsilosis* ATCC^®^ 22019, *Candida krusei* ATCC^®^ 6258 (currently *Pichia kudriavzevii*), and additionally, *Candida albicans* ATCC^®^ 90028 and *Candida albicans* ATCC^®^ 10231.

### 2.2. Fungal Susceptibility

Antifungal susceptibility results for rezafungin were interpreted primarily according to the EUCAST (v.12.0) guidelines, utilizing clinical breakpoints and Epidemiological Cut-off values (ECOFFs) where applicable. While CLSI-derived Epidemiological Cut-off Values (ECVs) are cited in the comparative tables for reference, they were not used for the primary classification of our isolates. All qualitative interpretations in this study strictly adhere to the EUCAST framework (ECOFF/breakpoint) to ensure methodological consistency.

The assessment of susceptibility to antifungal drugs currently available in Poland was performed using the Micronout-AM (BRUKER) test [17]. The performance, principle of operation, and interpretation of the test are consistent with the current guidelines of the EUCAST [18]. Drug susceptibility was assessed using microdilution, with antifungal drugs in 9–11 concentrations: amphotericin B (0.25–2 μg/mL); anidulafungin (0.002 μg/mL; 0.016–4 μg/mL); micafungin (0.002 μg/mL; 0.008–2 μg/mL); fluconazole (0.002 μg/mL; 0.5–32 μg/mL); posaconazole (0.03–4 μg/mL); voriconazole (0.03–4 μg/mL) in wells of a 96-well plate. Suspensions of the tested strains were prepared in Roswell Park Memorial Institute, Buffalo, NY, USA (RPMI-1640) supplemented with 3-(N-morpholino) propanesulfonic acid (MOPS) and 2% glucose (ThermoFisher Scientific, Waltham, MA, USA) and incubated on a microplate at 35 °C for 24–48 h in controlled environment conditions (according to the test manufacturer’s instructions). Afterward, the strain growth in subsequent test fields was assessed visually. The blue/purple color at the bottom indicated no growth, i.e., susceptibility to a given drug concentration. The change to pink or colorless indicated the strain growth, i.e., resistance to a given drug concentration. The assay was also performed for control strains recommended by the test manufacturer: *C. parapsilosis* ATCC 22019, *C. krusei* (currently *P. kudriavzevii*) ATCC 6258, and additionally, *C. albicans* ATCC 90028 and *Candida albicans* ATCC 10231.

### 2.3. Phenotypic Assay by Broth Microdilution Method (PMIC—The Planktonic Minimum Inhibitory Concentration)

The methodology for performing broth microdilution for amphotericin B and echinocandins followed the EUCAST E.Def 7.4 guidelines [19]. Namely, RPMI 1640 medium, supplemented with 2% glucose and buffered with 3-(N-morpholino)propanesulfonic acid (MOPS) (Merck, Darmstadt, Germany) at a final concentration of 0.165 mol/L (pH 7.0), served as the primary testing medium and contained Tween 20 (polysorbate 20) at 0.002%. Stock solutions of antifungal agents: amphotericin B (Y001361, Sigma-Aldrich, Burlington, MA, USA) and rezafungin (CAS 1631754-41-0, Angene International Limited, Hong Kong, China) were prepared using DMSO (Merck, Germany) according to ISO recommendation [20]. The highest concentration solution was diluted at least 200 times to ensure proper accuracy. A two-fold dilution series was prepared in RPMI 1640 medium to achieve the desired concentration range, 0.008–2 µg/mL for amphotericin B, and 0.002–1 µg/mL for rezafungin, following the schemes provided by EUCAST [18,19]. The antifungal concentrations were dispensed in 100 µL volumes into the first 11 columns of sterile flat-bottom 96-well microdilution. A yeast inoculum was prepared by suspending 5 distinct colonies from a 24 h culture in sterile distilled water, homogenizing the suspension, and adjusting its density to match a 0.5 McFarland standard, measured at 530 nm. A standardized yeast suspension, adjusted to 0.5–2.5 × 10^5^ CFU/mL, was added to each well to achieve a final concentration of 0.25–1.25 × 10^5^ CFU/mL. Column 12 was reserved as a growth control, containing 100 µ RPMI medium and 100 µL yeast suspension, and 1 well was left with medium only as a negative control. Once the plate was prepared, it was incubated at 35 ± 2 °C in ambient air without agitation. The results were read after 24 ± 2 h, using a spectrophotometer at 530 nm. For amphotericin B, the PMIC was defined as the lowest concentration inhibiting ≥90% growth compared to the drug-free control. For rezafungin, the PMIC was the lowest concentration inhibiting ≥ 50% growth compared to the drug-free control. If poor growth (absorbance ≤ 0.2) was observed, incubation was extended by 12–24 h before final readings. To ensure quality control, standard reference strains such as *C. parapsilosis* ATCC 22019 and *C. krusei* (currently *P. kudriavzevii*) ATCC 6258 were included in every test run. The MICs of these strains should decrease within the expected QC ranges, as defined by EUCAST. Results were compared with those of the Micronaut-AM (BRUKER) system. All PMIC determinations were performed in triplicate across three independent experiments to ensure consistency and reliability of the data. In the absence of established clinical breakpoints for rezafungin, isolates were categorized using Epidemiological Cut-off Values (ECOFFs). The rationale for this approach is to provide a standardized baseline for monitoring the susceptibility of clinical isolates in Poland. These values (0.25 μg/mL for *C. albicans* and 0.5 μg/mL for *N. glabratus* according to EUCAST) represent the upper limit of the MIC distribution for the wild-type population and are used here for epidemiological surveillance rather than for predicting individual clinical outcomes.

### 2.4. Crystal Violet Biofilm Assay

Biofilm biomass was quantified using the crystal violet (CV) assay. For each isolate, a suspension from an overnight culture on Sabouraud dextrose agar (SDA, bioMérieux Poland, Warsaw, Poland) was prepared in sterile distilled water and adjusted to 1 grade on the McFarland scale. The biofilm was grown in 24-well flat-bottom polystyrene plates (Costar, Corning Incorporated, Corning, NY, USA) in 1000 µL medium Sabouraud dextrose broth (SDB, BD BBL™, Franklin Lakes, NJ, USA) supplemented with 8% glucose. The plates were covered and incubated at 37 °C for 48 h. Each strain was analyzed in triplicate. Subsequently, the wells and unattached cells were rinsed 3 times using Dulbecco’s Phosphate-Buffered Saline (D-PBS) (Gibco, Carlsbad, CA, USA). Then, the plate was dried for 24 h upside down at room temperature. Then, 500 μL of 5% crystal violet solution (CV, Chempur, Karlsruhe, Germany) in 96% ethanol (Merck, Germany) was applied to each well and incubated for 15 min at room temperature. The solution was removed using a pipette, and the wells were washed twice with PBS solution.

Post-staining, absorbance was measured at 630 nm using a Tecan Infinite 200 PRO reader (Tecan Group Ltd., Männedorf, Switzerland). To account for potential heterogeneity in biofilm distribution, each well was scanned across a 12 × 12 grid (144 points), and the results were averaged. The optical density cut-off (A_c_) was defined as three standard deviations above the mean absorbance of the negative control (medium only). Based on the criteria established by Stepanović et al. [21], isolates were categorized as follows: non-adherent: A ≤ A_c_; weak biofilm producers: Ac < A ≤ 2 × A_c_; moderate biofilm producers: 2 × A_c_ < A ≤ 4 × A_c_; strong biofilm producers: A > 4 × A_c_, where A represents the average absorbance of the clinical isolate. The reference strain represented by *C. albicans* ATCC 90028 was used as a positive control.

### 2.5. Determination of Minimum Biofilm Inhibitory Concentration (MBIC) and Minimum Biofilm Elimination Concentration (MBEC)

In order to evaluate the rezafungin ability to inhibit biofilm development (MBIC) and eliminate biofilm (MBEC), the isolates were adhered to 96-well flat-bottom polystyrene plates in RPMI 1640 medium, containing L-glutamine, excluding bicarbonate (Gibco, Carlsbad, CA, USA), supplemented with 8% glucose (ThermoFisher Scientific, USA). Each microplate well was filled with 40 μL RPMI 1640 medium supplemented with 8% glucose, and then 10 μL of 1 × 10^7^ CFU/mL of tested clinical isolates was inoculated, resulting in a final density of 1 × 10^6^ CFU/mL. MBIC was determined on 96-well flat-bottomed microtiter polystyrene plates. To do this, the solutions of 50 μL rezafungin in the concentration range (25–0.05 μg/mL), diluted in RPMI 1640 medium supplemented with 8% glucose, were prepared. Subsequently, 50 μL of yeasts inoculums were added. After the 24 h incubation at 37 °C, the wells were rinsed 3 times with PBS (Gibco, Carlsbad, CA, USA), and the fresh medium (50 μL) with 10 μL resazurin (4 mg/mL) was added. Then, after 1 h incubation, MBIC was read, based on the visual color change from blue to pink and on the spectrophotometric reading. To determine the minimum biofilm eliminating concentration (MBEC), the plates were filled with 10 μL yeast isolate inoculum (1 × 10^7^ CFU/mL) in 90 μL RPMI 1640 medium supplemented with 8% glucose and incubated for 24 h at 37 °C. Subsequently, the wells were rinsed 3 times with PBS to remove non-adhered cells, and the fresh medium (100 μL) with a series of rezafungin concentrations (0.05–25 μg/mL) was added. After 24 h of incubation, 10 μL resazurin (4 mg/mL) was added to each well, and MBEC values were read. MBECs and MBICs were determined as the lowest concentration at which the resazurin reduction was lower than or equal to (10% ± 0.5%) either positive (100%) or negative (0%) controls using a spectrophotometer (OD_600_ value, Sunrise^TM^ Tecan, Männedorf, Switzerland). The metabolic activity was determined using the resazurin reduction assay (color change from blue to pink). All the experiments were conducted in triplicate [22].

### 2.6. Effect of Rezafungin on Biofilm Formation Using SEM

Scanning electron microscopy (SEM) was used to determine the rezafungin effect on the ability of *C. albicans* to form biofilm. A piece of sterile catheter (2 cm long) was placed in a 12-well cell culture cluster separately. To ensure uniform yeast adhesion, the sterile catheter was immersed in 1 mL of standardized cell suspension (1 × 10^7^ cells/mL) and incubated for 120 min at 37 °C in a shaker incubator (Biosan, Zielonka, Poland). To evaluate the morphological effects of rezafungin on biofilm architecture, catheters were exposed to three distinct concentrations: 25 µg/mL, 1.6 µg/mL, and 0.2 µg/mL. These concentrations were prepared in RPMI 1640 supplemented with 8% glucose. After 24 h of incubation at 37 °C, the samples were processed for imaging according to the methodology adapted from Alhede et al. [23]. Following the PBS washes, the catheter samples were fixed in 2.5% glutaraldehyde in 0.1 M phosphate buffer for 24 h at 4 °C. Subsequently, the samples were dehydrated through a graded ethanol series (30%, 50%, 70%, 80%, 90%, 95%, and 100% for 15 min each). Finally, the samples were dried at room temperature, mounted on aluminum stubs, and sputter-coated with carbon to ensure conductivity during imaging with the Tescan MIRA3 FEG-SEM (Tescan, Brno, Czech Republic) at the Jagiellonian Center of Innovation in Krakow.

### 2.7. Statistical Analysis

Statistical analyses were performed using the PQStat statistical package, version 1.8.4.152. A test probability of *p* < 0.05 was considered significant, and a test probability of *p* < 0.01 was considered highly significant. The different biofilm growth conditions and quantifications were tested in 3 replicates, and the standard deviation (SD) for each dataset was determined. The results of the analyzed scales, depending on the biofilm type, were compared using the Mann–Whitney U test (for two *C. albicans* groups) and the Kruskal–Wallis test (for three *N. glabratus* groups). The results of the analyzed scales, depending on the biofilm type, were analyzed using the Fisher exact test. The results of the analyzed scales, depending on “ECOFF” to “ECV”, were analyzed using the McNemar test. The comparison of MBEC to PMIC results was performed using the Wilcoxon test.

## 3. Results

The ultimate objective of this study is to enhance infection prevention within the vascular system, focusing on the potential antibiofilm activity of rezafungin. By characterizing the biofilm-forming ability and environmental adaptability of *C. albicans* and *N. glabratus* isolates, we aim to demonstrate how rezafungin disrupts early-stage colonization of medical devices. Its ability to inhibit β-(1,3)-D-glucan synthesis within the extracellular matrix is crucial for developing next-generation, biofilm-resistant vascular catheters and refining clinical protocols to prevent systemic dissemination. This approach builds upon our previous work on *Staphylococcus epidermidis* [16], shifting the focus toward the unique preventive challenges posed by eukaryotic fungal pathogens.

### 3.1. Antifungal Susceptibility Testing and ECOFF-Based Interpretation

In this study, the susceptibility of *C. albicans* and *N. glabratus* isolates to eight antifungal agents was assessed using the commercial Micronaut-AM test by determining the MIC value. The tested antifungal drugs included fluconazole (FLU), voriconazole (VOR), itraconazole (ITR), posaconazole (POS), amphotericin B (AMB), anidulafungin (AND), micafungin (MYC), and caspofungin (CAS). The MIC values indicated varying levels of susceptibility across the isolates (Table 1, Tables S1 and S3, Figure S1).

All the *C. albicans* (100%, 27/27) isolates demonstrated susceptibility to azole (*incl*. fluconazole, voriconazole, itraconazole, and posaconazole). The MIC values for fluconazole ranged from 0.25 to 1 μg/mL, while voriconazole had MIC between 0.008 and 0.06 μg/mL. The MIC values for itraconazole were consistently 0.03 and 0.008 μg/mL, respectively. For amphotericin B, all the isolates (100%, 27/27) were susceptible, with MIC ranging from 0.125 to 0.5 μg/mL. Most isolates (96.3%, 26/27) were susceptible to anidulafungin, with MIC values of 0.002 to 0.03 μg/mL. However, resistance was observed in 3.7% (1/27) of the isolates, with the MIC value of 0.06 μg/mL. Similarly, micafungin demonstrated susceptibility in 81.5% (22/27) of the isolates, with MIC values between 0.016 and 0.03 μg/mL. However, resistance was detected in 18.5% (5/27) of the isolates, with the MIC values ranging from 0.06 to 0.125 μg/mL.

In this study, 86.7% (13/15) *N. glabratus* strains were classified in the range of susceptible, increased exposure to fluconazole, while 13.3% (2/15) were resistant. The MIC values for fluconazole ranged from 0.5 to 128 μg/mL, reflecting reduced activity of this azole antifungal against *N. glabratus*. For voriconazole, itraconazole, and posaconazole, MIC values ranged from 0.008–1 µg/mL, 0.03–4 µg/mL, and 0.008–1 µg/mL, respectively. However, according to EUCAST guidelines, epidemiological cut-off values (ECV/ECOFF) for these antifungals were not established, preventing the isolates’ classification into susceptibility categories. In contrast, all the isolates (100%, 15/15) were susceptible to amphotericin B, with MIC values from 0.25 to 1 μg/mL. These findings highlight the continued efficacy of amphotericin B in treating *N. glabratus* infections. In this study, 100% of *N. glabratus* strains were susceptible to micafungin, whereas 1 out of 18 strains (5.6%) demonstrated resistance to anidulafungin, with MIC values from 0.016 to 0.06 µg/mL for both antifungal drugs.

For *C. albicans* and *N. glabratus*, the MIC range for caspofungin was 0.016–0.25 µg/mL and 0.06–0.25 µg/mL, respectively. According to EUCAST, the isolates susceptible to anidulafungin and micafungin should also be considered susceptible to caspofungin until specific breakpoints for caspofungin are established. Based on this rule, 16 out of 21 (76.2%) *C. albicans* isolates and 14 out of 15 (93.3%) *N. glabratus* isolates could be categorized as susceptible to caspofungin. However, 5 out of 21 (23.8%) *C. albicans* isolates and 1 out of 15 (6.7%) *N. glabratus* isolates could not be classified due to resistance to anidulafungin.

The classification of strains as “non-wild-type (NWT)” and “wild-type (WT)” strains according to the EUCAST [18,19] and CLSI guidelines [24] was summarized in Table S1 in the Supplementary Materials. The EUCAST method was used to determine MIC values for both compounds, so the interpretation in CLSI is not fully correct. Yet, some commercially available tests allow the MIC interpretation according to EUCAST and CLSI, despite using only one of the methods for its determination. The MIC values obtained for amphotericin B in both cases proved all the strains to be classified as WT. In the case of rezafungin, we noted a greater discrepancy. According to EUCAST, 55% of the strains were classified as NWT and 45% as WT. If the determined MIC values were to be qualified according to CLSI, 100% of strains would be considered WT (Table S2, Supplementary Materials).

The MICs determined by the CLSI and EUCAST methods generally demonstrated good correlation for both species with fluconazole and voriconazole. However, the EUCAST MICs are typically lower for amphotericin B, anidulafungin, micafungin, and posaconazole [25,26].

### 3.2. In Vitro Susceptibility of C. albicans and N. glabratus to Rezafungin

A total of 42 clinical isolates, comprising 27 *C. albicans* and 15 *N. glabratus*, were evaluated for their in vitro as WT to rezafungin. The MIC values were interpreted according to EUCAST species-specific epidemiological cut-off values (ECV/ECOFF) [18].

The MIC range for *C. albicans* isolates was 0.0039–0.0625 µg/mL. Among them, 9 isolates (33.3%) exhibited an MIC value of 0.0039, and 15 isolates (55.5%) exhibited 0.0156 µg/mL. Higher MIC values of 0.03125 µg/mL were observed in two isolates (7.4%) and 0.0625 µg/mL in one of them (3.7%). Therefore, with regard to *C. albicans*, 33.3% (9 isolates) were categorized as WT (MIC ≤ 0.008 µg/mL), while 66.7% (18 isolates) were NWT (MIC > 0.008 µg/mL).

Among 15 *N. glabratus* isolates, the MIC values in the dataset ranged from 0.0078 µg/mL to 0.03125 µg/mL. Based on the susceptibility threshold (i.e., MIC ≤ 0.016 µg/mL = susceptible; MIC > 0.016 µg/mL = resistant [16]), 66.7% (10 isolates) were classified as susceptible, and 33.3% (5 isolates) were classified as NWT (Supplementary Materials, Tables S1 and S2). This indicates that, while the majority of isolates were susceptible, a significant proportion was classified as NWT, posing challenges for antifungal treatment.

### 3.3. Biofilm Forming Ability of Both Species

Table S4 in the Supplementary Materials presents the summary of three readings for biofilms produced by the examined isolates. The values resulted from subtracting the absolute absorbance value, i.e., the value of 0.095 obtained for the negative control (medium without inoculum). The reference strain *C. albicans* ATCC 90028, with a proven ability to produce biofilm, served as a positive control. Its mature biofilm value was 0.445. The maximum absorbance (A) value of the biomass formed after staining it with crystal violet was 0.716 for *C. albicans* and 0.496 for *N. glabratus*. The minimum value for both species was 0.03 (average 0.31 ± 0.03).

According to the criteria described by Stepanović et al. [21], 42 tested strains were biofilm producers of varying intensity. The results showed that 26 strains (62%) were strong biofilm producers. Moderate and weak biofilm producers comprised 14% (*n* = 6) and 24% (*n* = 10), respectively, in the *N. glabratus* group. The strains were classified in terms of particular species in Table 2.

**Figure 1:**
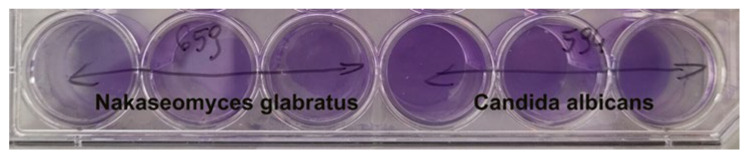
CV method; 24-well working plate—evaluation of biofilm production capacity for *N. glabratus* no. 659 and C. *albicans* no. 594. Both strains were characterized by strong biofilm production (mean A_630_ = 0.472 and 0.384, respectively).

The tested strains were capable of forming biofilms under experimental conditions, but with various levels of such a capacity. Namely, *C. albicans* strains proved a strong ability to produce biofilm, while most of the *N. glabratus* strains showed a moderate and weak one. However, when analyzing the results obtained by microtiter plate and CV methods, we found a similar trend in biomass formation in different clinical strains (Figure 1, Table S4 in Supplementary Materials). The rank scale for biofilm production determined through crystal violet staining revealed that *C. albicans* produced significantly greater amounts of biofilm than *N. glabratus* (*p* < 0.05).

### 3.4. Planktonic (PMIC) and Biofilm Susceptibility Testing (MBIC and MBEC Values)

In accordance with the EUCAST yeast critical points, PMICs of rezafungin and amphotericin B for *C. albicans* in comparison to MBICs and MBECs were found to be statistically significant (Table S6, Supplementary Materials). Furthermore, based on the statistical analysis, a significant positive correlation (Mann–Whitney U test *p* = 0.0178) was found with PMIC of amphotericin B increasing in the commercial Micronaut-AM assay in the *C. albicans* isolates of a higher biofilm formation capacity. Anidulafungin, micafungin, and caspofungin showed no significant differences (*p* > 0.05). There was no significant (Mann–Whitney U test *p* = 0.3008) difference in the results for *N. glabratus.*

The strain’s ability to inhibit biofilm formation and eradicate mature biofilm was rated by determining MBIC and MBEC for the selected antifungal drugs (amphotericin B) and the new echinocandin, rezafungin. The results are tabulated for 26 clinical isolates, including only 21 of *C. albicans* and 5 of *N. glabratus* ones endowed with a strong ability to produce biofilm (Table S5, Supplementary Materials).

**Figure 2:**
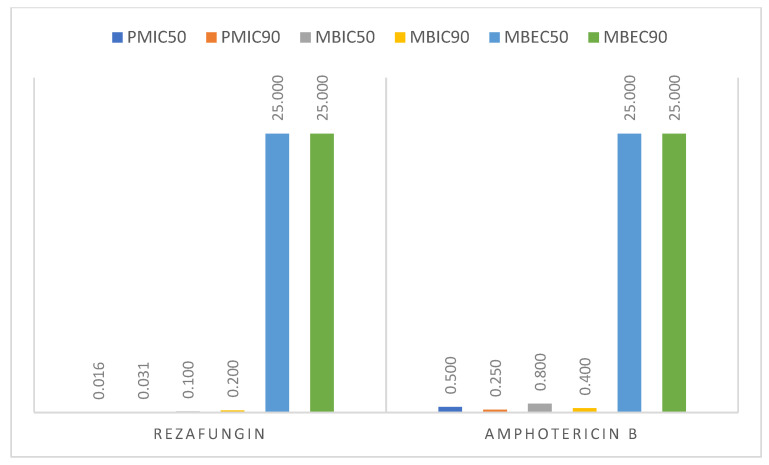
Summary of PMIC_50_, PMIC_90_, MBIC_50_, MBIC_90_, MBEC_50_, and MBEC_90_ values for *Candida* strains and antifungal drugs.

Rezafungin and amphotericin B showed comparable degrees of mature biofilm eradication (MBEC = 25 µg/mL). The MBIC values were 10–100 times, and MBECs were 1000 times higher than PMIC for rezafungin, which is characteristic of yeast forms concentrated in biofilms (Figure 2).

Comparison of the obtained PMIC_50_ and PMIC_90_ values for rezafungin confirmed its greater in vitro activity (MIC_50_ values = 0.016 µg/mL and MIC_90_ = 0.031 µg/mL) than the amphotericin B activity (MIC_50_ values = 0.5 µg/mL and MIC_90_ = 0.25 µg/mL).

### 3.5. Biofilm Scanning Electron Microscopy (SEM)

**Figure 3:**
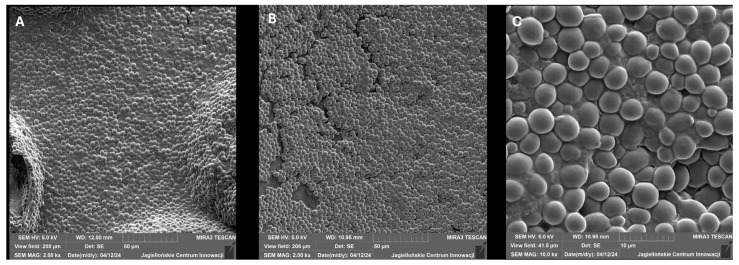
Clinical strain *C. albicans* no. 579—biofilm formed on fragment of central venous catheter (CVC). Representative scanning electron microscopy (SEM) micrograph of a mature *Candida albicans* biofilm. The image illustrates a complex, three-dimensional network consisting of both yeast-phase cells and elongated hyphal structures. The cells are interconnected and embedded within a visible layer of extracellular polymeric substances (EPSs), which forms a protective matrix. Magnification: 2.00 kx; accelerating voltage: 5.0 kV. Panels (**A**) and (**B**) show the macrostructure of the biofilm network (scale bars = 50 µm). Panel (**C**) presents a high-magnification view of individual cells and extracellular matrix (scale bar = 10 µm).

The aim of the conducted experiment was to assess *C. albicans*, clinical strain no. 579, in terms of biofilm formation on the medical device surface, namely, on a fragment of a central catheter. Figure 3 shows the sample photos of biofilm produced by *C. albicans* with a confirmed strong ability to form biofilm (average value of A_630_ = 0.716 obtained in the microtiter plate method).

**Figure 4:**
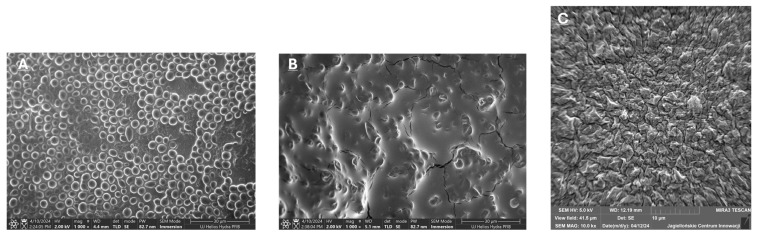
SEM micrograph of Candida albicans biofilm treated with rezafungin. Sector (**A**)—strain culture in medium without rezafungin; (**B**,**C**)—yeast cells changed by rezafungin. A significant reduction in biofilm biomass and architectural disintegration is observed. The treatment resulted in the collapse of the hyphal network and visible damage to the fungal cell walls (cell wall shriveling). The extracellular polymeric substance (EPS) matrix is sparse and fragmented compared to the control group. Magnification: 2.00 kx; scale bar: 30 and 10 µm.

In addition, the effect of rezafungin at various concentrations on the examined strain cells was illustrated. The results for the clinical strain *C. albicans* no. 576 with a confirmed strong ability to produce biofilm, and based on the obtained values (PMIC = 0.04 µg/mL and MBEC = 25 µg/mL), are presented in Figure 4.

The ability of *Candida* isolates to form biofilms was visually confirmed using crystal violet staining (Figure 1). The photographs demonstrate a consistent and dense layer of biomass adhering to the surfaces, with *C. albicans* and *N. glabratus* showing intense staining, indicative of high biofilm productivity. This macro-scale visualization aligns with our quantitative findings and underscores the persistent nature of these pathogens in vascular-like environments. These results, combined with the microscopic details from SEM (Figure 3 and Figure 4), provide a comprehensive overview of the biofilm-forming strategies employed by these fungi to resist infection prevention measures.

## 4. Discussion

The global burden of fungal infections is underscored by the 2022 World Health Organization (WHO) designation of *C. auris* and *C. albicans* as critical-priority pathogens in the Fungal Priority Pathogens List [27]. A major therapeutic challenge stems from the increasing prevalence of *Candida* biofilms on medical devices and host tissues. Biofilm formation is a key virulence factor that promotes high levels of antifungal resistance, necessitating the urgent development of effective antifungals with antibiofilm activity.

The development of next-generation antifungal agents, such as rezafungin—a novel echinocandin with an extended half-life—represents a global advancement in treating invasive candidiasis and is not restricted to any specific geographic region [1]. While the primary metabolic targets of such compounds (e.g., β-1,3-glucan synthase) are highly conserved across *Candida albicans* strains worldwide, the pharmacological response can exhibit regional variability. Studies have shown that, while the core metabolic framework remains identical, local strains may develop specific mutations (e.g., in the *FKS* genes) or compensatory metabolic shunts in response to regional antifungal prescribing patterns [27]. Therefore, while rezafungin acts through a universal mechanism, monitoring local susceptibility profiles, such as those in Poland, remains critical for optimizing therapeutic outcomes. While *C. albicans* remains the most prevalent pathogen, the clinical significance of non-albicans species, particularly *N. glabratus*, is increasing in Polish hospitals. From a pharmacological perspective, *N. glabratus* often exhibits reduced susceptibility to fluconazole, making the introduction of newer agents like rezafungin even more critical. In our study, the Bruker Micronaut-AM system was used for susceptibility testing. While automated systems are widely adopted in routine clinical laboratories, it is crucial to interpret MIC values derived from commercial assays in the context of reference methods (CLSI, EUCAST), as they may show varying degrees of essential or categorical agreement. The role of the Micronaut-AM system in susceptibility testing must be interpreted alongside its known limitations. It has been documented that some commercial platforms may produce higher MIC values for echinocandins than the EUCAST reference broth microdilution method. To address this, our study included a direct comparison between the Micronaut-AM system and the broth microdilution methodology. While the commercial system provides practical advantages in a clinical laboratory setting, the potential for MIC overestimation was carefully considered during our data analysis. By correlating these two methods, we aimed to provide a more nuanced interpretation of the susceptibility rates, acknowledging that any observed “MIC creep” might be partially attributed to the testing platform itself. Regarding *C. albicans*, only 33.3% of isolates were classified as WT to rezafungin, having MIC values below 0.008 µg/mL, in line with the EUCAST classification. Conversely, the majority (66.7%) exhibited MIC > 0.008 µg/mL. This high proportion of NWT strains contrasts sharply with large-scale surveillance data: A robust estimate from the SENTRY Program (using CLSI reference methods) demonstrated that rezafungin was highly active, with 98.5% of *C. albicans*, *C. tropicalis*, *N. glabratus*, and *C. krusei* isolates inhibited at an ECV 0.12 µg/mL [28]. Specifically, for *C. albicans*, MIC_50_/_90_ was 0.03/0.06 µg/mL [29]. A Nordic study [30] (using the EUCAST reference method) similarly reported high activity, MIC_50/90_ = 0.06 µg/mL, and classified only 1.9% of *C. albicans* isolates as NWT. The significant discrepancy between our high non-susceptibility rate and these reference studies may be attributed to the Micronaut-AM system. Our high MIC value (0.0156 µg/mL) for *C. albicans* may reflect this methodological variance. The clinical relevance of these findings is supported by Soriano et al. [31], who conducted a pooled analysis of two randomized trials (STRIVE and REMISSION) comparing rezafungin with caspofungin. They noted that reduced susceptibility of *C. albicans* to rezafungin raises concerns about the drug’s efficacy, particularly where NWT isolates were involved. Crucially, in a pre-specified sensitivity analysis of isolates categorized as NWT to rezafungin (in vitro), the success rate dropped significantly to 40% (2/5 isolates). This marked reduction in efficacy for NWT strains strongly validates the need to carefully interpret even slightly elevated MIC values, like the 0.0156 µg/mL value observed in our study, in light of potential clinical failures. Turning to *Nakaseomyces glabratus* (formerly *C. glabrata*), the observed 33.3% NWT to rezafungin in our isolates is clinically relevant due to the species’ intrinsic azole resistance and its association with echinocandin resistance via glucan synthase gene (*FKS1*) mutations. The observed differences in rezafungin efficacy between *C. albicans* and *N. glabratus* might be linked to structural and functional variations in their primary pharmaceutical targets. Rezafungin, like other echinocandins, inhibits the 1,3-β-D-glucan synthase enzyme. Discrepancies in susceptibility are often associated with mutations in the “hot spot” regions of the *FKS1* and *FKS2* genes. In *N. glabratus*, the upregulation of *FKS2* is a well-documented mechanism of reduced susceptibility, which may explain the higher MBEC values observed in our study. Moreover, the effectiveness of antifungals can be influenced by the complexity of the biofilm matrix and the specific metabolic pathways of the species involved, a phenomenon also observed in studies of various bioactive compounds where inter-species discrepancies are common. The observed discrepancies in rezafungin effectiveness across different *Candida* species may be linked to specific pharmaceutical targets. As reported in recent studies on antifungal mechanisms [32], the susceptibility of *Candida* spp. is often determined by the drug’s ability to inhibit the yeast-to-hyphae morphological transition and disrupt membrane integrity. Specifically, agents affecting the synthesis of membrane ergosterol can induce membrane depolarization and lipid peroxidation, leading to a critical loss of intracellular material such as proteins and DNA. Furthermore, the induction of reactive oxygen species (ROS) levels following treatment provides an additional layer of structural damage. In our study, the lower susceptibility of *N. glabratus* compared to *C. albicans* might be attributed to differences in these membrane-related targets and the inherent inability of *N. glabratus* to form true hyphae, which alters the biofilm’s architectural response to echinocandins.

While SENTRY surveillance reported high WT rates for rezafungin in *N. glabratus* [29], with ECV 0.12 µg/mL [28], studies comparing automated systems with reference methods for this species and echinocandins have shown varying degrees of agreement. A key advantage of rezafungin is its pharmacokinetic profile. The ECV values determined in the SENTRY study are far below the peak plasma concentrations of 22 to 30 µg/mL achievable at the 400 mg dose [28].

Furthermore, MIC values for *fks* mutant strains of *C. albicans* (0.25 µg/mL) and *N. glabratus* (2 µg/mL) are within concentrations estimated to achieve 100% probability of PK-PD target attainment through week 6 [28]. Given that the high plasma drug exposure of rezafungin easily exceeds the Mutant Prevention Concentration (MPC) of 16 µg/mL for *Candida*, a possible advantage of rezafungin may be to prevent the development of resistance to the echinocandin class of antifungal agents [33].

Our results concerning comparator agents reinforce current therapeutic considerations. Fluconazole exhibited reduced activity against *N. glabratus* (86.7% intermediate, 13.3% resistant), consistent with known azole resistance in this species. Conversely, amphotericin B (100% susceptible) and micafungin (100% susceptible) were highly effective. These findings are broadly consistent with the work of Al-Baqsami et al. [34], whose analysis reported high susceptibility to micafungin and amphotericin B, while showing significant non-susceptibility to fluconazole. This is also reinforced by the SENTRY data [28], which showed that species identification alone should be used cautiously as the sole criterion for anti-*Candida* agent selection due to emerging fluconazole resistance in species traditionally considered susceptible [35,36]. We acknowledge that the rates of reduced susceptibility to rezafungin observed in our study, particularly regarding *C. albicans*, are higher than those reported in large-scale global surveillance programs. These discrepancies may be attributed to the specific methodology employed in our research or the characteristics of our local isolate collection. Consequently, our findings should be regarded as preliminary and method-dependent rather than indicative of a broad epidemiological shift. Further studies using standardized reference methods are necessary to validate these observations and to determine whether they reflect emerging local resistance patterns or technical variations.

The therapeutic challenge posed by biofilms is further highlighted by the intrinsic resistance of azoles and conventional amphotericin B against *Candida* biofilms. Echinocandins offer further potential therapeutic applicability, demonstrating both safety and efficacy against biofilms by targeting 1,3-β-glucan synthesis to control extracellular matrix (ECM) production [32].

Our structural analysis using the crystal violet (CV) assay confirmed a species-dependent biofilm capacity: *C. albicans* > *N. glabratus*, consistent with the findings of Marcos-Zambrano et al. [37]. The minimum biofilm eradication concentrations (MBEC_50_ and MBEC_90_) for rezafungin were determined to be 25 µg/mL for the majority of the tested strains, indicative of mature biofilm resistance. The profound increase in resistance compared to planktonic cells is a well-documented phenomenon [33,38,39], primarily attributed to the EPS matrix, efflux pumps, and persister cells. In contrast to mature biofilm, we found that rezafungin exhibited some antifungal activity against young biofilm, depending on the species. Chandra et al. [14] demonstrated that rezafungin possessed antibiofilm activity against both adhesion-phase and mature-phase forms of *C. albicans*. Our results proved rezafungin to suppress biofilm formation within minutes of the treatment initiation, disrupting/deforming adhering cells and preventing further biofilm development. Our SEM observations for rezafungin reveal structural damage similar to that reported for other echinocandins, such as micafungin or anidulafungin, which typically cause cell wall collapse and hyphal fragmentation. However, rezafungin’s stability and prolonged half-life may offer superior prevention of biofilm regrowth on catheter surfaces compared to traditional agents, as suggested by our MBEC results. It is crucial to emphasize that the MBEC values observed in this study were significantly higher than the corresponding planktonic MICs, often by several orders of magnitude. From a clinical perspective, such high concentrations typically exceed the maximum plasma concentration (C_max_) achievable with standard dosing of rezafungin [29]. Therefore, these in vitro findings should be interpreted as a characterization of the drug’s anti-biofilm activity under experimental conditions rather than a direct indication of therapeutic potential. While the high stability and long half-life of rezafungin are advantageous, the eradication of established biofilms in a clinical setting remains a significant challenge that may not be fully addressed by monotherapy at currently approved doses. Despite these findings, several limitations of our study must be acknowledged. First, the research was conducted as a single-center study with a limited number of isolates, which may not fully represent the broader epidemiological landscape or regional variations in susceptibility patterns. Consequently, the results should be viewed as preliminary and specific to our local clinical setting. Future multicenter studies involving a larger and more diverse collection of isolates are necessary to confirm these observations and to provide a more comprehensive understanding of rezafungin activity against *Candida albicans* and *Nakaseomyces glabratus* biofilms.

### Limitations of This Study

Despite the clinical importance of our findings, several limitations of this study must be acknowledged. First, this was a single-center study with a relatively small sample size (*n* = 42), which reflects its pilot nature. Consequently, the results may not fully represent the nationwide epidemiology of *C. albicans* and *N. glabratus* in Poland. Second, as clinical breakpoints for rezafungin have not yet been established by EUCAST, our interpretations are based on proposed ECOFF values rather than validated clinical outcomes. Additionally, while the in vitro biofilm models provide valuable insights into antifungal activity, they may not fully replicate the complex environmental conditions and host immune responses present during systemic infections. Future large-scale, multicenter investigations are necessary to validate these preliminary findings and to establish the long-term clinical efficacy of rezafungin against biofilm-associated vascular infections.

## 5. Conclusions

This preliminary study provides original data on the antifungal activity of rezafungin against a specific collection of clinical isolates from vascular infections. Our results revealed that a fraction of the tested isolates, particularly *C. albicans*, exhibited reduced susceptibility in the Micronaut-AM system, suggesting potential variability between commercial and reference methodologies. This highlights the importance of routine susceptibility testing, as species identification may not always be a sufficient predictor of the resistance profile. Furthermore, while mature biofilms showed significant resistance to rezafungin (high MBEC values), the drug demonstrated a promising ability to suppress early-stage biofilm formation. This suggests a potential role for rezafungin as a preventative agent in device colonization, although further studies with a larger and more diverse pool of isolates are required to confirm these findings and explore the underlying molecular mechanisms, such as mutations in the *FKS* gene subunits. However, the elevated MBEC values compared to planktonic MICs highlight the inherent resilience of fungal biofilms. These results, while promising, are strictly limited to laboratory observations and should not be directly extrapolated to clinical outcomes. Further research, including in vivo models and clinical trials, is essential to determine the practical therapeutic relevance of these findings in the management of biofilm-related infections. These results represent a pilot study of regional importance, providing a baseline for future surveillance of rezafungin activity in Poland.

## Figures and Tables

**Table 1:** Distributions of MIC_50_ and MIC_90_ values for isolates of *C. albicans* and *N. glabratus* in commercial panel (MICRONAUT-AM).

	*C. albicans*			*N. glabratus*		
	MIC_50_	MIC_90_	Range	MIC_50_	MIC_90_	Range
FLU	0.5	0.5	0.25–1	8	16	0.5–128
VOR	0.008	0.008	0.008–0.06	0.06	0.125	0.008–1
ITR	0.03	0.03	0.03	0.125	4	0.03–4
POS	0.08	0.08	0.08	0.25	0.5	0.008–1
AMB	0.25	0.5	0.125–0.5	0.5	0.5	0.25–1
AND	0.016	0.03	0.0016–0.06	0.03	0.03	0.016–0.06
MYC	0.016	0.016	0.016–0.125	0.016	0.016	0.016–0.06
CAS	0.06	0.125	0.016–0.25	0.125	0.25	0.06–0.25

FLU—fluconazole, VOR—voriconazole, ITR—itraconazole. POS—posaconazole, AMB—amphotericin B, AND—anidulafungin, MYC—micafungin, CAS—caspofungin.

**Table 2:** Biofilm production by *C. albicans* and *N. glabratus* isolates according to criteria interpretation by Stepanović.

Average A Value Biofilm Production	*N* (%)		Biofilm Formation
	*C. albicans*	*N. glabratus*	
A ≤ 0.095 (A ≤ Ac)	0 (0%)	0 (0%)	Non-biofilm producer
0.095 < A ≤ 0.19 (Ac < A ≤ 2 × Ac)	6 (22%)	4 (27%)	Weak biofilm producer
0.19 < A ≤ 0.38 (2 × Ac < A ≤ 4 × Ac)	0 (0%)	6 (40%)	Moderate biofilm producer
0.38 < A (4 × Ac < A)	21 (78%)	5 (33%)	Strong biofilm producer
Ac = 0.095			

N—overall frequency; %—percentage.

## Data Availability

The original contributions presented in this study are included in the article/Supplementary Materials. Further inquiries can be directed to the corresponding authors.

## Supplementary Materials

The following supporting information can be downloaded at: https://www.mdpi.com/article/10.3390/pharmaceutics18020213/s1, Table S1: Epidemiological cut-off values (ECOFFs) and available clinical breakpoints for *C. albicans* and *N. glabratus* yeasts using EUCAST and CLSI methodologies. Isolates were classified as wild-type (WT) or non-wild-type (NWT) based on the indicated ECOFF/ECV values; Table S2: Summary of phenotypic categorization for rezafungin and amphotericin B based on ECOFF and ECV values in the broth microdilution method; Table S3: Distribution of MIC values of *Candida* and *Nakaseomyces* isolates by commercial test (MICRONAUT-AM) and broth microdilution method (PMIC); Table S4: Summary of the results of the phenotypic assessment of biofilm production by the tested *C. albicans* and *N. glabratus* strains obtained using the microtitre plate method; Table S5: PMIC values versus MBIC and MBEC values (μg/mL) results of rezafungin and amphotericin B for *Candida* and *Nakaseomyces* strains; Table S6: Comparison of PMIC, MBIC and MBEC values for rezafungin and amphotericin B; Figure S1: Graphical presentation of sensitivity test results for individual antifungal drugs. FLU—fluconazole; ITR—itraconazole; VOR—voriconazole; POS—posaconazole; AND—anidulafungin; MYC—micafungin; AMB—amphotericin B; REZ—rezafungin.

## Abbreviations

The following abbreviations are used in this manuscript:

MIC	Minimum inhibitory concentration
MBIC	Minimum biofilm inhibitory concentration
MBEC	Minimum biofilm eradication concentration
PMIC	Planktonic minimum inhibitory concentration
QC	Quality control
IFIs	Invasive fungal infections
EMA	European Medicines Agency
FDA	US Food and Drug Administration
PK/PD	Pharmacokinetic and pharmacodynamic
MALDI-TOF MS	Matrix-associated laser desorption ionization time-of-flight mass spectrometry
EUCAST	European Committee on Antimicrobial Susceptibility Testing
CLSI	Clinical and Laboratory Standards Institute
RPMI-1640	Roswell Park Memorial Institute
MOPS	3-(N-morpholino) propanesulfonic acid
ATCC	American Type Culture Collection
DMSO	Dimethyl sulfoxide
CFU	Colony-forming unit
SDA	Sabouraud dextrose agar
CV	Crystal violet
OD	Optical density
SEM	Scanning electron microscope
FLU	Fluconazole
VOR	Voriconazole
ITR	Itraconazole
POS	Posaconazole
AMB	Amphotericin B
AND	Anidulafungin
MYC	Micafungin
CAS	Caspofungin
A	Absorbance
CVC	Central venous catheter
WT	Wild-type
NWT	Non-wild-type
ECV	Epidemiological cut-off values CLSI
ECOFF	Epidemiological cut-off values EUCAST
ECM	Extracellular matrix
MPC	Mutant prevention concentration

## References

[1] Rayens E., Norris K.A. (2022). Prevalence and Healthcare Burden of Fungal Infections in the United States, 2018. Open Forum Infect. Dis..

[2] Bongomin F., Gago S., Oladele R.O., Denning D.W. (2017). Global and Multi-National Prevalence of Fungal Diseases-Estimate Precision. J. Fungi.

[3] Kidd S.E., Abdolrasouli A., Hagen F. (2023). Fungal Nomenclature: Managing Change is the Name of the Game. Open Forum Infect. Dis..

[4] Wang J.F., Xue Y., Zhu X.B., Fan H. (2015). Efficacy and safety of echinocandins versus triazoles for the prophylaxis and treatment of fungal infections: A meta-analysis of RCTs. Eur. J. Clin. Microbiol. Infect. Dis..

[5] Sherry L., Ramage G., Kean R., Borman A., Johnson E.M., Richardson M.D., Rautemaa-Richardson R. (2017). Biofilm-Forming Capability of Highly Virulent, Multidrug-Resistant *Candida auris*. Emerg. Infect. Dis..

[6] Rezzayo (Rezafungin) EPAR. https://www.ema.europa.eu/en/medicines/human/EPAR/rezzayo#overview.

[7] US FDA (2023). REZZAYO (Rezafungin for Injection): Prescribing Information.

[8] Krishnan B.R., James K.D., Polowy K., Bryant B.J., Vaidya A., Smith S., Laudeman C.P. (2017). CD101, a novel echinocandin with exceptional stability properties and enhanced aqueous solubility. J. Antibiot..

[9] Sharma D., Vazquez J.A. (2024). An evaluation of Rezafungin: The latest treatment option for adults with candidemia and invasive candidiasis. Expert. Opin. Pharmacother..

[10] Garcia-Effron G. (2020). Rezafungin-Mechanisms of Action, Susceptibility and Resistance: Similarities and Differences with the Other Echinocandins. J. Fungi.

[11] Ham Y.Y., Lewis J.S., Thompson G.R. (2021). Rezafungin: A novel antifungal for the treatment of invasive candidiasis. Future Microbiol..

[12] Rubino C.M., Flanagan S. (2021). Population Pharmacokinetics of Rezafungin in Patients with Fungal Infections. Antimicrob. Agents Chemother..

[13] Thompson G.R., Soriano A., Cornely O.A., Kullberg B.J., Kollef M., Vazquez J., Honore P.M., Bassetti M., Pullman J., Chayakulkeeree M. (2023). Rezafungin versus caspofungin for treatment of candidaemia and invasive candidiasis (ReSTORE): A multicentre, double-blind, double-dummy, randomised phase 3 trial. Lancet.

[14] Chandra J., Ghannoum M.A. (2018). CD101, a Novel Echinocandin, Possesses Potent Antibiofilm Activity against Early and Mature *Candida albicans* Biofilms. Antimicrob. Agents Chemother..

[15] Malinovská Z., Čonková E., Váczi P. (2023). Biofilm Formation in Medically Important Candida Species. J. Fungi.

[16] Skiba-Kurek I., Nowak P., Empel J., Tomczak M., Klepacka J., Sowa-Sierant I., Żak I., Pomierny B., Karczewska E. (2021). Evaluation of Biofilm Formation and Prevalence of Multidrug-Resistant Strains of Staphylococcus epidermidis Isolated from Neonates with Sepsis in Southern Poland. Pathogens.

[17] Bruker MICRONAUT-AM. https://www.bruker.com/en/products-and-solutions/microbiology-and-diagnostics/antimicrobial-susceptibility-testing/micronaut-am.html.

[18] European Committee on Antimicrobial Susceptibility Testing (2024). Breakpoint Tables for Interpretation of MICs Version 11.0, Valid from 2024-02-12.

[19] EUCAST Method for the Determination of Broth Dilution Minimum Inhibitory Concentrations of Antifungal Agents for Yeasts. The European Committee on Antibiotic Susceptibility Testing v 7.4, Valid from 13 October 2023. https://www.eucast.org/eucast_news/news_singleview?tx_ttnews%5Btt_news%5D=558&cHash=2e3a6144f491f0fbc6e828b0f58791a6.

[20] (2019). Susceptibility Testing of Infectious Agents and Evaluation of Performance of Antimicrobial Susceptibility Test Devices—Part 1: Broth Micro-Dilution Reference Method for Testing the In Vitro Activity of Antimicrobial Agents Against Rapidly Growing Aerobic Bacteria Involved in Infectious Diseases.

[21] Stepanović S., Vuković D., Hola V., Di Bonaventura G., Djukić S., Ćirković I., Ruzicka F. (2007). Quantification of biofilm in microtiter plates: Search for optimal conditions. APMIS.

[22] Jaśkiewicz M., Janczura A., Nowicka J., Kamysz W. (2019). Methods Used for the Eradication of Staphylococcal Biofilms. Antibiotics.

[23] Alhede M., Qvortrup K., Liebrechts R., Høiby N., Givskov M., Bjarnsholt T. (2012). Combination of microscopic techniques reveals a comprehensive visual impression of biofilm structure and composition. FEMS Immunol. Med. Microbiol..

[24] (2017). Reference Method for Broth Dilution Antifungal Susceptibility Testing of Yeasts.

[25] Arendrup M.C., Prakash A., Meletiadis J., Sharma C., Chowdhary A. (2017). Comparison of EUCAST and CLSI Reference Microdilution MICs of Eight Antifungal Compounds for *Candida auris* and Associated Tentative Epidemiological Cutoff Values. Antimicrob. Agents Chemother..

[26] Pfaller M.A., Castanheira M., Messer S.A., Rhomberg P.R., Jones R.N. (2014). Comparison of EUCAST and CLSI broth microdilution methods for the susceptibility testing of 10 systemically active antifungal agents when tested against *Candida* spp.. Diagn. Microbiol. Infect. Dis..

[27] WHO (2022). WHO Fungal Priority Pathogens List to Guide Research, Development and Public Health Action.

[28] Pfaller M.A., Carvalhaes C., Messer S.A., Rhomberg P.R., Castanheira M. (2020). Activity of a Long-Acting Echinocandin, Rezafungin, and Comparator Antifungal Agents Tested against Contemporary Invasive Fungal Isolates (SENTRY Program, 2016 to 2018). Antimicrob. Agents Chemother..

[29] Bassetti M., Stewart A., Bartalucci C., Vena A., Giacobbe D.R., Roberts J. (2025). Rezafungin acetate for the treatment of candidemia and invasive candidiasis: A pharmacokinetic evaluation. Expert. Opin. Drug Metab. Toxicol..

[30] Helleberg M., Jørgensen K.M., Hare R.K., Datcu R., Chowdhary A., Arendrup M.C. (2020). Rezafungin In Vitro Activity against Contemporary Nordic Clinical Candida Isolates and *Candida auris* Determined by the EUCAST Reference Method. Antimicrob. Agents Chemother..

[31] Soriano A., Locke J.B., Cornely O.A., Roilides E., Ramos-Martinez A., Honoré P.M., Castanheira M., Carvalhaes C.G., Nseir S., Bassetti M. (2025). Clinical and mycological outcomes of candidaemia and/or invasive candidiasis by *Candida* spp. and antifungal susceptibility: Pooled analyses of two randomized trials of rezafungin versus caspofungin. Clin. Microbiol. Infect..

[32] Zorić N., Kosalec I., Tomić S., Bobnjarić I., Jug M., Vlainić T., Vlainić J. (2017). Membrane of Candida albicans as a target of berberine. BMC Complement. Altern. Med..

[33] Larkin E.L., Dharmaiah S., Ghannoum M.A. (2018). Biofilms and beyond: Expanding echinocandin utility. J. Antimicrob. Chemother..

[34] Al-Baqsami Z.F., Ahmad S., Khan Z. (2020). Antifungal drug susceptibility, molecular basis of resistance to echinocandins and molecular epidemiology of fluconazole resistance among clinical *Candida glabrata* isolates in Kuwait. Sci. Rep..

[35] Singh A., Healey K.R., Yadav P., Upadhyaya G., Sachdeva N., Sarma S., Kumar A., Tarai B., Perlin D.S., Chowdhary A. (2018). Absence of Azole or Echinocandin Resistance in *Candida glabrata* Isolates in India despite Background Prevalence of Strains with Defects in the DNA Mismatch Repair Pathway. Antimicrob. Agents Chemother..

[36] Khalifa H.O., Arai T., Majima H., Watanabe A., Kamei K. (2020). Genetic Basis of Azole and Echinocandin Resistance in Clinical *Candida glabrata* in Japan. Antimicrob. Agents Chemother..

[37] Marcos-Zambrano L.J., Escribano P., Bouza E., Guinea J. (2016). Comparison of the antifungal activity of micafungin and Micronaut-AM against *Candida tropicalis* biofilms. J. Antimicrob. Chemother..

[38] Marzucco A., Gatti G., Montanari M.S., Fantini M., Colosimo C., Tamburini M.V., Arfilli V., Morotti M., Schiavone P., Congestrì F. (2024). Evaluation of Biofilm Production and Antifungal Susceptibility to Fluconazole in Clinical Isolates of *Candida* spp. in Both Planktonic and Biofilm Form. Microorganisms.

[39] Melo A.S., Bizerra F.C., Freymüller E., Arthington-Skaggs B.A., Colombo A.L. (2011). Biofilm production and evaluation of antifungal susceptibility amongst clinical *Candida* spp. isolates, including strains of the *Candida parapsilosis* complex. Med. Mycol..

