# Supplementary material for: In Vitro Activity of Rezafungin Against Planktonic and Biofilm Forms of Candida albicans and Nakaseomyces glabratus Clinical Isolates from Vascular Infections in Poland: A Pilot Study
Source: Pharmaceutics. 2026 Feb 8;18(2):213. doi: 10.3390/pharmaceutics18020213 (PMC12943897; doi:10.3390/pharmaceutics18020213)
Supplement: Supplementary file 1 [file pharmaceutics-18-00213-s001.zip › pharmaceutics-4111373-supplementary.pdf]

pharmaceutics

Supporting Informations

In Vitro Activity of Rezafungin against Planktonic and Biofilm Forms of *Candida albicans* and *Nakaseomyces glabratus* Clinical Isolates from Vascular Infections in Poland: A Pilot Study

Iwona Skiba-Kurek[1], Magdalena Namysł[1*], Katarzyna Kania[2,3,] Joanna Czekajewska[2], Anna Sepioło[1], Tomasz Gosiewski[4], Aldona Olechowska-Jarząb[1*]

Table S1. Epidemiological cut-off values (ECOFFs) and available clinical breakpoints for *C. albicans* and *N. glabratus* yeasts using EUCAST and CLSI methodologies. Isolates were classified as Wild-Type (WT) or Non-Wild-Type (NWT) based on the indicated ECOFF/ECV values.

		EUCAST (mμg/mL)				CLSI (μg/mL)				
**Species**	**Drug**	**S (≤)**	**I**	**R (>)**	**ECOFF**	**S (≤)**	**I**	**SDD**	**R (≥)**	**ECV***
Candida albicans	**AMB**	1		1	1	-	-	-	-	2
	**AND**	0.016		0.016	0.016	0.25	0.5	-	1	0.12
	**MIC**	0.03		0.03	0.03	0.25	0.5	-	1	0.03
	**REZ**	0.008		0.008	0.125	0.25	-	-	-	0.06
	**FLU**	2	4	4	0.5	2	-	4	8	0.5
	**ITR**	0.06		0.06	0.03	-	-	-	-	-
	**POS**	0.06		0.06	0.06	-	-	-	-	0.06
	**VOR**	0.06	0.125–0.25	0.25	0.03	0.12	0.25–0.5	-	1	0.03
	**ISA**	IE		IE						

https://doi.org/10.3390/pharmaceutics18020213

***Nakaseomyces glabratus* (formerly *Candida glabrata*)**	**AMB**	1		1	1	-	-	-	-	2
	**AND**	0.06		0.06	0.06	0.12	0.25	-	0.5	0.25
	**MIC**	0.03		0.03	0.03	0.06	0.12	-	0.25	0.03
	**REZ**	0.016		0.016	0.25	0.5	-	-	-	0.12
	**FLU**	0.001	≤16	16	16	-	-	≤32	64	8
	**ITR**	-	-	-	2	-	-	-	-	4
	**POS**	-	-	-	1	-	-	-	-	1
	**VOR**	-	-	-	1	-	-	-	-	0.25
	**ISA**	-	-	-	ND					

AMB – amphotericin B, AND – anidulafungin, MIC – micafungin, REZ – rezafungin, FLU – fluconazole, VOR – voriconazole, ITR – itraconazole, POS – posaconazole, VOR – voriconazole, ISA – isavuconazole, ECOFF – EUCAST – epidemiological cut-off value, ECV – CLSI epidemiological cut-off value, ND - Not Determined / No Data, IE - Insufficient Evidence, SDD - Susceptible, Dose-Dependent

*CLSI ECVs are provided for comparative purposes only; primary interpretation is based on EUCAST ECOFFs/breakpoints

Table S2. Summary of phenotypic categorization for rezafungin and amphotericin B based on ECOFF and ECV values in the broth microdilution method.

**Rezafungin ECOFF (EUCAST)**						
**NWT**	4 (66.67%)	0 (%)	17 (80.95%)	1 (25%)	2 (33.33%)	2 (40%)
**WT**	2 (33.33%)	0 (%)	4 (19.05%)	3 (75%)	4 (66.67%)	3 (60%)
**Fisher's exact test (*p*=)**	0.5875			1.0000		
**Rezafungin ECV (CLSI)**						
**NWT**	0 (0%)	0 (0%)	0 (0%)	0 (0%)	0 (0%)	0 (0%)
**WT**	6 (100%)	0 (0%)	21 (100%)	4 (100%)	6 (100%)	5 (100%)
**Fisher's exact test (*p*=)**	----			----		
**Amphotericin B ECOFF (EUCAST)**						

**NWT**	0 (%)	0 (%)	0 (%)	0 (%)	0 (%)	0 (%)
**WT**	6 (100%)	0 (%)	21 (100%)	4 (100%)	6 (100%)	5 (100%)
**Fisher's exact test (*p*=)**	----					
**Amphotericin B ECV (CLSI)**						
**NWT**	0 (0%)	0 (0%)	0 (0%)	0 (0%)	0 (0%)	0 (0%)
**WT**	6 (100%)	0 (0%)	21 (100%)	4 (100%)	6 (100%)	5 (100%)
**Fisher's exact test (*p*=)**	----			----		

Table S3. Distribution of MIC values of *Candida* and *Nakaseomyces* isolates by commercial test (MICRONAUT-AM) and broth microdilution method (PMIC).

							**MICRONAUT-AM**				**PMIC**	
								**MIC (µg/mL)**				
**No.**	**Strain**	**Source**	**FLU**	**VOR**	**ITR**	**POS**	**AMB**	**AND**	**MYC**	**CAS**	**Rezafungin**	**Amphotericin B**
**1**	*C. albicans*	CVC	0.5	0.06	0.03	0.008	0.5	0.016	0.06	0.125	0.016	0.25
**2**	*C. albicans*	CVC	0.5	0.008	0.03	0.008	0.5	0.06	0.03	0.125	0.016	0.5
**3**	*C. albicans*	blood	0.5	0.008	0.03	0.008	0.125	0.016	0.016	0.06	0.016	0.25
**4**	*C. albicans*	blood	0.5	0.008	0.03	0.008	0.25	0.016	0.016	0.06	0.03	0.25
**5**	*C. albicans*	blood	0.5	0.008	0.03	0.008	0.25	0.03	0.06	0.125	0.06	0.25
**6**	*C. albicans*	blood	0.5	0.008	0.03	0.008	0.5	0.03	0.125	0.125	0.004	0.5
**7**	*C. albicans*	blood	0.25	0.008	0.03	0.008	0.25	0.03	0.016	0.06	0.016	0.25
**8**	*C. albicans*	blood	0.25	0.008	0.03	0.008	0.25	0.03	0.016	0.06	0.03	0.25
**9**	*C. albicans*	blood	0.25	0.008	0.03	0.008	0.25	0.016	0.016	0.06	0.016	0.25
**10**	*C. albicans*	blood	1	0.008	0.03	0.008	0.25	0.016	0.016	0.06	0.004	0.25

**11**	*C. albicans*	blood	0.5	0.008	0.03	0.008	0.5	0.016	0.016	0.125	0.016	0.5
**12**	*C. albicans*	blood	0.5	0.016	0.03	0.008	0.5	0.002	0.016	0.125	0.016	0.5
**13**	*C. albicans*	CVC	0.5	0.008	0.03	0.008	0.25	0.03	0.016	0.06	0.016	0.25
**14**	*C. albicans*	blood	0.5	0.008	0.03	0.008	0.5	0.016	0.016	0.125	0.016	0.5
**15**	*C. albicans*	blood	0.5	0.008	0.03	0.008	0.5	0.016	0.016	0.06	0.016	0.5
**16**	*C. albicans*	blood	0.5	0.008	0.03	0.008	0.25	0.016	0.016	0.06	0.004	0.5
**17**	*C. albicans*	CVC	1	0.008	0.03	0.008	0.25	0.03	0.016	0.125	0.004	0.25
**18**	*C. albicans*	CVC	0.5	0.008	0.03	0.008	0.25	0.002	0.016	0.06	0.016	0.25
**19**	*C. albicans*	CVC	1	0.008	0.03	0.008	0.25	0.03	0.016	0.125	0.004	0.25
**20**	*C. albicans*	CVC	0.5	0.008	0.03	0.008	0.5	0.03	0.06	0.125	0.016	0.5
**21**	*C. albicans*	blood	0.5	0.008	0.03	0.008	0.5	0.002	0.016	0.016	0.016	0.25
**22**	*C. albicans*	CVC	0.25	0.008	0.03	0.008	0.25	0.016	0.016	0.125	0.004	0.25
**23**	*C. albicans*	blood	0.5	0.008	0.03	0.008	0.5	0.03	0.016	0.125	0.004	0.5
**24**	*C. albicans*	CVC	0.5	0.008	0.03	0.008	0.25	0.03	0.016	0.06	0.016	0.25
**25**	*C. albicans*	blood	0.5	0.008	0.03	0.008	0.25	0.03	0.016	0.125	0.016	0.25
**26**	*C. albicans*	blood	0.5	0.008	0.03	0.008	0.25	0.016	0.016	0.06	0.004	0.25
**27**	*C. albicans*	blood	0.5	0.008	0.03	0.008	0.25	0.002	0.016	0.06	0.004	0.25
**28**	*N. glabratus*	blood	8	0.03	0.03	0.016	0.25	0.016	0.016	0.25	0.03	0.25
**29**	*N. glabratus*	blood	0.5	0.008	0.03	0.008	0.25	0.016	0.016	0.06	0.016	0.25
**30**	*N. glabratus*	blood	64	1	4	1	0.5	0.06	0.016	0.125	0.03	0.25
**31**	*N. glabratus*	blood	4	0.016	0.03	0.008	0.5	0.016	0.016	0.125	0.03	0.5
**32**	*N. glabratus*	blood	8	0.06	2	0.25	0.5	0.06	0.016	0.25	0.016	0.25
**33**	*N. glabratus*	blood	8	0.06	0.125	0.125	0.5	0.03	0.016	0.125	0.016	0.5
**34**	*N. glabratus*	blood	16	0.125	4	0.5	0.5	0.03	0.016	0.06	0.008	0.25

**35**	*N. glabratus*	blood	128	1	4	1	1	0.03	0.016	0.125	0.03	0.5
**36**	*N. glabratus*	CVC	16	0.25	0.5	0.25	0.5	0.03	0.016	0.06	0.016	0.5
**37**	*N. glabratus*	blood	4	0.06	0.125	0.25	0.5	0.03	0.016	0.125	0.016	0.25
**38**	*N. glabratus*	blood	16	0.125	0.25	0.125	0.5	0.03	0.06	0.125	0.03	0.5
**39**	*N. glabratus*	blood	8	0.125	0.25	0.125	1	0.03	0.016	0.125	0.016	0.5
**40**	*N. glabratus*	CVC	8	0.06	0.06	0.125	0.5	0.03	0.016	0.25	0.016	0.5
**41**	*N. glabratus*	blood	8	0.06	0.125	0.25	0.5	0.03	0.016	0.125	0.016	0.5
**42**	*N. glabratus*	CVC	8	0.06	0.25	0.125	0.25	0.016	0.016	0.06	0.016	0.25

*fluconazole (FLU), voriconazole (VOR), itraconazole (ITR), posaconazole (POS), amphotericin B (AMB), anidulafungin (AND), micafungin (MYC), and caspofungin (CAS)

Table S4. Summary of the results of the phenotypic assessment of biofilm production by the tested *C. albicans* and *N. glabratus* strains obtained using the microtitre plate method.

**No.**		**Strain**	**I**	**II**	**III**	**Mean OD [630 nm]**	**SD**	**ODc**	**Biofilm formation**
**1**	608	*C. albicans*	0.448	0.446	0.542	0.479	0.055	0.643	strong
**2**	566	*C. albicans*	0.392	0.317	0.325	0.345	0.041	0.468	strong
**3**	556	*C. albicans*	0.095	0.087	0.097	0.093	0.005	0.109	weak
**4**	579	*C. albicans*	0.433	0.367	0.418	0.406	0.035	0.51	strong
**5**	588	*C. albicans*	0.572	0.623	0.463	0.553	0.082	0.798	strong
**6**	600	*C. albicans*	0.486	0.485	0.475	0.482	0.006	0.5	strong
**7**	551	*C. albicans*	0.636	0.639	0.621	0.632	0.01	0.661	strong
**8**	553	*C. albicans*	0.122	0.144	0.112	0.126	0.016	0.175	weak

**9**	681	*C. albicans*	0.584	0.659	0.602	0.615	0.039	0.732	strong
**10**	526	*C. albicans*	0.713	0.726	0.708	0.716	0.009	0.744	strong
**11**	471	*C. albicans*	0.33	0.291	0.267	0.296	0.032	0.391	strong
**12**	475	*C. albicans*	0.311	0.353	0.37	0.345	0.03	0.436	strong
**13**	544	*C. albicans*	0.121	0.21	0.243	0.191	0.063	0.381	strong
**14**	594	*C. albicans*	0.322	0.327	0.353	0.334	0.017	0.384	strong
**15**	664	*C. albicans*	0.356	0.343	0.297	0.332	0.031	0.425	strong
**16**	712	*C. albicans*	0.394	0.357	0.316	0.356	0.039	0.473	strong
**17**	645	*C. albicans*	0.318	0.306	0.362	0.329	0.029	0.417	strong
**18**	597	*C. albicans*	0.324	0.354	0.368	0.349	0.022	0.416	strong
**19**	535	*C. albicans*	0.063	0.08	0.097	0.08	0.017	0.131	weak
**20**	598A	*C. albicans*	0.533	0.475	0.469	0.492	0.035	0.598	strong
**21**	591B	*C. albicans*	0.383	0.456	0.513	0.451	0.065	0.646	strong
**22**	715	*C. albicans*	0.412	0.393	0.382	0.396	0.015	0.441	strong
**23**	687	*C. albicans*	0.306	0.301	0.353	0.32	0.029	0.406	strong
**24**	668	*C. albicans*	0.625	0.598	0.594	0.606	0.017	0.656	strong
**25**	688	*C. albicans*	0.139	0.101	0.105	0.115	0.021	0.178	weak
**26**	694	*C. albicans*	0.121	0.125	0.109	0.118	0.008	0.143	weak
**27**	611	*C. albicans*	0.121	0.115	0.12	0.119	0.003	0.128	weak
**28**	607	*N. glabratus*	0.236	0.227	0.22	0.228	0.008	0.252	moderate
**29**	581	*N. glabratus*	0.216	0.146	0.14	0.167	0.042	0.294	moderate
**30**	527	*N. glabratus*	0.276	0.253	0.203	0.244	0.037	0.356	moderate
**31**	634	*N. glabratus*	0.449	0.515	0.523	0.496	0.041	0.618	strong
**32**	533	*N. glabratus*	0.156	0.1	0.113	0.123	0.029	0.211	moderate
**33**	563	*N. glabratus*	0.158	0.128	0.176	0.154	0.024	0.227	moderate
**34**	534	*N. glabratus*	0.476	0.472	0.464	0.471	0.006	0.489	strong
**35**	659	*N. glabratus*	0.372	0.309	0.269	0.317	0.052	0.472	strong

**36**	665	*N. glabratus*	0.119	0.089	0.088	0.099	0.018	0.152	weak
**37**	542	*N. glabratus*	0.274	0.227	0.204	0.235	0.036	0.342	strong
**38**	567	*N. glabratus*	0.138	0.132	0.134	0.135	0.003	0.144	weak
**39**	658	*N. glabratus*	0.124	0.113	0.116	0.118	0.006	0.135	weak
**40**	536	*N. glabratus*	0.158	0.128	0.11	0.132	0.024	0.205	moderate
**41**	707	*N. glabratus*	0.108	0.104	0.092	0.101	0.008	0.126	weak
**42**	598B	*N. glabratus*	0.422	0.338	0.29	0.35	0.067	0.55	strong
	C. albicans ATCC 90028		0.465	0.431	0.439	0.445	0.018	0.498	strong

Table S5. PMIC values versus MBIC and MBEC values (µg/mL) results of rezafungin and amphotericin B for *Candida* and *N. glabratus* selected isolates.

No.		**PMIC (µg/mL)**		**MBIC (µg/mL)**		**MBEC (µg/mL)**	
		**Rezafungin**	**Amphotericin B**	**Rezafungin**	**Amphotericin B**	**Rezafungin**	**Amphotericin B**
1	608	0.016	0.25	0.005	0.4	25	25
2	566	0.004	0.5	0.005	0.2	25	25
3	579	0.004	0.25	0.005	0.4	25	25
4	588	0.004	0.25	0.005	0.4	25	25
5	600	0.004	0.5	0.1	0.4	25	25
6	551	0.004	0.25	0.1	0.4	25	25
7	681	0.004	0.25	0.1	0.4	25	25
8	526	0.008	0.25	0.1	0.4	25	25
9	471	0.016	0.5	0.1	0.2	25	25

10	475	0.016	0.5	0.1	0.2	25	25
11	544	0.016	0.25	0.1	0.4	25	25
12	594	0.016	0.5	0.1	1.6	25	25
13	664	0.016	0.5	0.1	0.8	25	25
14	712	0.016	0.5	0.2	0.4	25	25
15	645	0.016	0.25	0.2	0.4	25	25
16	597	0.016	0.25	0.2	0.2	25	25
17	598A	0.016	0.5	0.2	0.8	25	25
18	591B	0.016	0.25	0.2	0.4	25	25
19	715	0.016	0.25	0.2	0.8	25	25
20	687	0.016	0.5	0.2	0.4	25	25
21	668	0.016	0.25	0.2	0.4	25	25
22	634	0.016	0.5	0.2	0.8	25	25
23	534	0.016	0.25	0.2	0.4	25	25
24	659	0.031	0.5	0.2	0.4	25	25
25	542	0.031	0.25	0.4	0.8	25	25
26	598B	0.031	0.25	0.4	0.8	25	25
	C. albicans ATCC 90028	0.063	0.25	0.4	0.8	25	25

Table S6. Comparison of PMIC, MBIC and MBEC values for rezafungin and amphotericin B.

Strains		*C. albicans*			*N. glabratus*		
Biofilm formation		MBIC	MBEC	PMIC	MBIC	MBEC	PMIC
Wilcoxon test for rezafungin	Z	3.9064	4.1233	----	1.0787	1.9052	----
	p	**0.0001**	**<0.0001**	----	0.2807	0.0568	----
Wilcoxon test for amfotericin B	Z	2.0077	4.1256		1.6255	1.9322	
	p	**0.0447**	**<0.0001**		0.1041	0.0533	

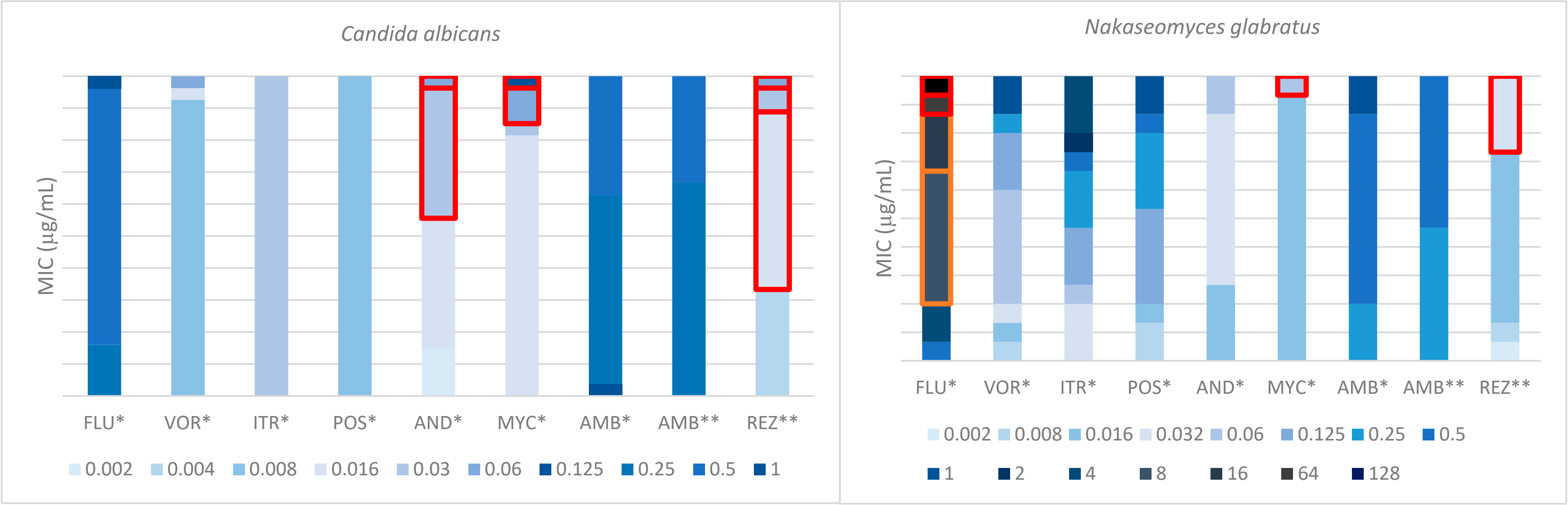

Figure S1. Graphical presentation of sensitivity test results for individual antifungal drugs. FLU – fluconazole; ITR – itraconazole; VOR – voriconazole; POS – posaconazole; AND - anidulafungin; MYC – micafungin; AMB – amphotericin B; REZ – rezafungin.

* - test was performed using Micronaut-AM test

** - test was performed using microdilution method in RPMI medium

red frame – resistant; orange frame – susceptible, increased exposure
